# Natural language analyzed with AI-based transformers predict traditional subjective well-being measures approaching the theoretical upper limits in accuracy

**DOI:** 10.1038/s41598-022-07520-w

**Published:** 2022-03-10

**Authors:** Oscar N. E. Kjell, Sverker Sikström, Katarina Kjell, H. Andrew Schwartz

**Affiliations:** 1 Department of Psychology, Lund University, Lund, Sweden; 2 Department of Computer Science, Stony Brook University, Stony Brook, USA

**Keywords:** Psychology, Human behaviour

## Abstract

We show that using a recent break-through in artificial intelligence –*transformers–*, psychological assessments from text-responses can approach theoretical upper limits in accuracy, converging with standard psychological rating scales. Text-responses use people's primary form of communication –*natural language*– and have been suggested as a more ecologically-valid response format than closed-ended rating scales that dominate social science. However, previous language analysis techniques left a gap between how accurately they converged with standard rating scales and how well ratings scales converge with themselves – a theoretical upper-limit in accuracy. Most recently, AI-based language analysis has gone through a transformation as nearly all of its applications, from Web search to personalized assistants (e.g., Alexa and Siri), have shown unprecedented improvement by using transformers. We evaluate transformers for estimating psychological well-being from questionnaire text- and descriptive word-responses, and find accuracies converging with rating scales that approach the theoretical upper limits (Pearson *r* = 0.85, *p* < 0.001, *N* = 608; in line with most metrics of rating scale reliability). These findings suggest an avenue for modernizing the ubiquitous questionnaire and ultimately opening doors to a greater understanding of the human condition.

Words are the natural medium with which a person expresses their state of mind. However, in personality and social psychology today, research is dominated by asking participants to express themselves in the form of numeric rating scales where complex states of mind are represented by predefined answers from a rating scale. A typical social and psychological article uses 20 measures on average, approximately 87% of which are closed-ended numeric rating scales^1^. Although rating scales have contributed to important findings in social and personality psychology and other fields, they come with drawbacks. A person asked to communicate their inner thoughts and emotions in response to a question (e.g., *Are you satisfied with your life?*), normally provides a descriptive, open-ended answer in words (e.g., *I’m very satisfied. Most of my expectations are met…*), rather than closed-ended numeric or category-based answers (e.g., *6* = *Agree*).

Language reflects, for example, our personality^2,3^, daily emotions^4^, mental health^5,6^, and behaviors^7,8^. As such, “language is the most common and reliable way for people to translate their internal thoughts and emotions into a form that others can understand. Words and language, then, are the very stuff of psychology and communication”^9^. This power of language to reflect psychological aspects of a person, is beginning to be possible to quantify. Recent evaluations show that psychologically meaningful scores can be produced by artificial intelligence (AI)-based language assessments^5,10^. Anywhere a survey is being used, respondents could instead be asked to freely describe their mental state rather than forced to adhere to closed-ended responses^10^.

Thus, as an alternative to rating scales, questionnaire language-based assessments involve measuring mental states with open-ended responses that are quantified and analyzed with techniques from AI^10^. The questionnaire language-based assessments provide structure in its prompt, while also allowing respondents to *freely* describe their state of mind. Research shows that asking respondents to answer with descriptive word-responses or free text-responses, can be used to predict corresponding rating scales^10^. This was done in seven studies using answers related to satisfaction with life and harmony in life (Pearson’s *r* = 0.72, *p* < 0.001). Although this method is promising, some hesitation in the adoption of language-based assessments exists due to an *accuracy gap*. Language-based assessments’ correlations to rating scales have fallen short of the accuracy degree with which rating scales can be trusted (i.e., taking into account their reliability, measurement error) – which can be seen as a theoretical upper-limit of possible alignment to a rating scale.

Recent advances in AI-based text analysis have yielded unprecedented performance gains in many traditional applications of AI such as web search, automatic machine translation or question answering. These advances are attributed to a new machine learning technique referred to as the *transformer*^11^. Transformers are large, general purpose statistical models that have been shown to capture the meaning of words in their context^11,12^; for example, it understands the difference between “I feel *great*” and “it was a *great* personal loss”. To date the most widely cited transformer-language-model is *BERT* (Bidirectional Encoder Representations from Transformers)^12^. Evaluations over a variety of standard AI-tests including language classification tasks (i.e., not psychological assessments tasks) demonstrated that the model typically resulted in 10% or greater reductions in error compared to earlier models^12^. Being able to represent the different meanings of a word depending on its context, can enable researchers to better capture the meaning of what a person is trying to express.

Building on a wide tradition in the literature of subjective well-being^13–15^, we focus on the satisfaction with life scale^16^ as our measure of well-being. Further, to demonstrate our results replicate to newer subjective well-being constructs we also test against the Harmony in Life Scale^17^. Most often the cognitive component of subjective well-being is assessed through satisfaction with life, which encourages life evaluations that are based on comparing one’s actual with one’s desired life circumstances^16^. More recently subjective well-being has also been assessed through harmony in life, which emphasizes the relational aspect of well-being^18^ and encourages life evaluations that consider one’s interconnectedness with other aspects of one's life^17,19^. Satisfaction with life and harmony in life have been found to meaningfully complement each other in capturing a more comprehensive understanding of well-being^17,19–21^.

Assessing subjective well-being is particularly suitable with open-ended responses because it concerns how an individual subjectively (and potentially uniquely) thinks about their life (i.e., their life evaluations). The subjective aim to well-being emphasizes that respondents should be able to consider unique aspects of what they find is important and meaningful regarding their evaluations^13,22^. Whereas, the closed-ended rating scale format requires respondents to evaluate fixed rating scales and thus does not allow the generation of unique evaluations, this is achieved with open-ended responses. However, despite advantages of natural language – the difficulties of quantifying it have previously resulted in imperfect accuracy.

Here, we examine whether *transformers* (i.e., BERT) can close the accuracy gap and bring language-based assessments closer to a theoretical upper-limit of accuracy as compared to rating scales. We further examine different aspects of the language-based assessment method to understand how they may be used to modernize surveys and the way we understand the human condition. In short, the current analyses involve applying numeric representations (called *word embeddings*) from pre-trained language models to quantify respondents’ word- and text-responses; and then training these word embeddings to predict the rating scales using multiple linear regression. The accuracy is measured as the Pearson correlation between predicted and observed scores using cross-validation (described in the Methods section).

## Results

### Language-based assessments as accurate as rating scales’ reliability

The reliability measures for the Harmony in life scale included *r* = 0.76 for the mean of inter-item correlations, and *r* = 0.84 for the mean of item-total correlations; and its previously demonstrated test–retest reliability range from *r* = 0.71 to 0.77^10,17^. For the Satisfaction with life scale the mean of the inter-item correlations was *r* = 0.73, and the mean of the item-total correlations *r* = 0.82; and the previously demonstrated test–retest reliabilities ranged from *r* = 0.82 to 0.84^10,17^.

In theory, the reliability of a measure represents a maximum correlation one might expect to that specific measure given the noise of the measure^23^. Thus, we take the 0.71–0.84 reliability scores for the Harmony in life scale, and the 0.73–0.84 for the Satisfaction with life scale to define an upper-limit of how accurately an alternative measure could expect to converge with these measures, given the noise of the measures. The language-based assessments from all word- and text-responses (i.e., responses to questions about both harmony in life and satisfaction with life) using contextualized word embeddings predict the rating scale rivaling its reliability measures. Observed and predicted Harmony in life scale scores yield a very strong Pearson correlation of 0.85 (*p* < 0.001; Table 1), which is significantly stronger than the mean of inter-item correlations, stronger than the test–retest reliability measures and in line with the mean of item-total correlations. The correlation between predicted and observed scores for the Satisfaction with life scale also yields a strong correlation of *r* = 0.80, which is here significantly stronger than its inter-item correlation, and approximately in line with its test–retest and the mean of item-total correlations. Descriptive statistics and correlations among the numeric variables are presented in Table SM1 and SM2.

**Table 1:** Comparing Pearson Correlations based on All Responses Combined and Analyzed with Contextualized Word Embeddings to the Reliability of the Rating Scales.

Model	HILS	SWLS
BERT contextualized word embeddings from word- and text-responses of HIL and SWL	0.85^↑^***	0.80^↑^**
**Reliability measures**		
Inter-item Pearson correlation average	0.76	0.73
Corrected item-total Pearson correlation average	0.84	0.82
*Test–retest reliability1*^10^	*0.71*	*0.82*
*Test–retest reliability2*^17^	*0.77*	*0.84*

Italic values indiactes results from other articles/datasets.

All correlations were significant at *p* < 0.001. *N* = 608.

HIL = Harmony in life; SWL = Satisfaction with life; S = Scale.

^**↑**^ = significantly higher than Inter-item correlation average; *** = *p* < 0.001, ** = *p* < 0.001.

### Current language-based assessments improve previous state-of-the-art

The current language-based assessments for both Harmony in life and Satisfaction with life significantly improve upon the previous state-of-the-art based on a context-free language model and only one word-response format. Using the previous state-of-the-art method for Harmony in life yields a Pearson *r* of 0.75 (Table 2); hence our new correlation produces a significant (*p* < 0.001) increase of 13%. For Satisfaction with life, the previous method produces an r of 0.72 , where our new method yields a significant (*p* < 0.001) increase of 11%^c.f.^^10^.

**Table 2:** Comparison of Pearson Correlations Using Contextualized versus Decontextualized Word Embeddings for Individual Word- and Text-Responses.

Word Embeddings		Text-response		Word-response	
		HIL	SWL	HIL	SWL
		HILS	SWLS	HILS	SWLS
Context	BERT	0.74	0.74	0.79	0.75
No context	BERT 1 word/docs	0.54^↓^	0.59^↓^	0.78	0.75
	Latent Semantic Analysis	0.47^↓^	0.46^↓^	0.75^↓^	0.72

All correlations were significant at *p* < .001. *N* = 608.

HIL = Harmony in life; SWL = Satisfaction with life; S = Scale.

Latent Semantic Analysis based on Google 5-gram, 512 dimensions; number of dimensions were optimized as described in^10^ (i.e., based on previous state-of-the-art).

^↓^ = significantly smaller than BERT. See Table SM3 for more comparisons.

### Language-based assessments can distinguish well-being dimensions

To be able to differentiate between concepts, it is important that a measure has noticeably lower correlations with measures from which it theoretically differs. This important characteristic of a psychological measure is called *discriminant validity*. Even though the Harmony in life scale and the Satisfaction with life scale correlate very strongly (*r* = 0.85), both Harmony in life word- and text-responses predict the *corresponding* rating scale the Harmony in life scale (*r* = 0.79 and 0.74) significantly more accurately than the Satisfaction with life scale (*r* = 0.66 and 0.61; Table. 3; word-responses: bootstrapped *p* = 0.003; text-responses: bootstrapped *p* = 0.004). But neither Satisfaction with life word- nor text-responses predict the Satisfaction with life scale significantly better than the Harmony in life scale (word-responses: *r* = 0.75 versus 0.75, bootstrapped *p* = 0.877; text-responses: *r* = 0.74 versus 0.71, bootstrapped *p* = 0.284).

**Table 3:** The Construct Specific Validity of Language Models Using Individual Word- and Text-Responses.

Language Model	Predict	Pearson r			
		Harmony in life responses		Satisfaction with life responses	
		Words	Text	Words	Text
BERT	HILS	0.79^↑^	0.74^↑^	0.75	0.71
	SWLS	0.66	0.61	0.75	0.74

*N* = 608. HIL = Harmony in life; SWL = Satisfaction with life; S = Scale.

^↓^ = significantly higher than SWLS prediction.

Whereas, the Harmony in life scale is not significantly more accurately predicted by Harmony in life words as compared with Satisfaction with life words (*r* = 0.79 versus 0.75, *p* = 0.064), it is significantly more accurately predicted by Harmony in life text-responses rather than Satisfaction with life text-responses (*r* = 0.74 versus 0.71, *p* = 0.013). And the Satisfaction with life scale is more accurately predicted by the Satisfaction with life word-responses rather than the Harmony in life word-responses (*r* = 0.75 versus 0.66, *p* < 0.001) and Satisfaction with life text-responses rather than Harmony in life text-responses (*r* = 0.74 versus 0.61, *p* < 0.001).

Further, even though the predicted Harmony in life scale and the predicted Satisfaction with life scale scores are very strongly correlated (*r* = 0.96, *p* < 0.001; Table 4), it is possible to train the language models to *differentiate* between the two with significant accuracy. This is achieved by training the word embeddings to predict the difference scores of the rating scales (i.e., the normalized Harmony in life scale-scores minus the normalized Satisfaction with life scale-scores). The highest accuracy is achieved using all responses, which yield considerable accuracy (*r* = 0.34, *p* < 0.001), especially considering the strong correlation between the Harmony in life scale and the Satisfaction with life scale (*r* = 0.85, *p* < 0.001).

**Table 4:** The Discriminant Validity of Language Models: Significantly Predicting the Harmony in life scale Minus the Satisfaction with life scale.

Language Model	Responses	Predicted HILS correlated with Predicted SWLS	Accuracy (*r*) of Predicted HILS minus SWLS
BERT	All	0.96	0.34
	Words	0.97	0.25
	Text	0.96	0.27

*N* = 608. All correlations (Pearson r) were significant at *p* < .001.

*Accuracy (r) of Predicted HILS minus SWLS* = predicting the difference score of the normalized HILS minus the normalized SWLS, where normalization was achieved by respectively subtracting the column mean from each score.

### Contextualized word embeddings best for text response predictions

Next we investigate to what extent different aspects of the language-based assessments contribute to its validity. An important research question concerns whether word- or text-response formats are most suitable for capturing mental states. Previous research shows that descriptive words rather than text-responses yield more accurate predictions of rating scales (word-responses: *r* = 0.72; text-responses: *r* = 0.49)^10^. However, those algorithms were unable to capture the word order (i.e., a context-free language model). Here, contextualized BERT embeddings are compared with decontextualized embeddings by employing BERT context-free and a context-free non-transformer model previously used for language-based assessments referred to as Latent Semantic Analysis^24^.

The contextualized word embeddings produce substantial increments for text-responses compared to when the context is removed. Contextualized BERT significantly increases the predictive accuracy of text-responses as compared to both BERT decontextualized and Latent Semantic Analysis embeddings (Table 2). Compared to the previous state-of-the-art (i.e., Latent Semantic Analysis), contextualized word embeddings produce significantly higher accuracy for both the Harmony in life scale (*r* = 0.74 versus 0.47, *p* < 0.001) and the Satisfaction with life scale (*r* = 0.74 versus 0.46, *p* < 0.001).

The contextualized embeddings did not create substantial improvements for word responses, where there was only a significant difference between predictions of the Harmony in life scale from Harmony in life word-responses based on BERT versus Latent Semantic Analysis (0.79 versus 0.75, *p* < 0.01; for Satisfaction with life word-responses: 0.75 versus 0.72, *p* = 0.056).

### Word responses produce somewhat higher accuracy than text responses

Analyses based on all words versus all text responses analyzed with BERT demonstrate that word-responses produce more accurate predictions for the Harmony in life scale (0.83 versus 0.79, *p* = 0.002; Table 5), but not for the Satisfaction with life scale (0.77 versus 0.75, *p* = 0.540). That word-responses overall tend to produce a slightly higher accuracy in some situations is also reflected in the information content that the responses carry. There is more information in the word- (Diversity Index = 874.5) as compared with the text-responses (Diversity Index = 409.4). This means that word responses, although containing fewer words, contain greater amount of the mathematical concept of *information*; in other words, if one was to store these two pieces of data in their most efficient forms, the word-responses would require more bits on the computer (i.e. they would take up more disk space) than the text-responses even though the text-responses were much longer. That the words from the word-responses comprise the highest information is consistent with the results showing that they produce slightly higher correlations, since more information gives the machine learning algorithms more information to use.

**Table 5:** Word- versus Text-Responses: Accuracy (r), Diversity Index, and Mean (SD) number of words.

Language Response	r, HILS prediction	r, SWLS prediction	Diversity Index of Words Input	Mean (SD) of N words
HIL + SWL Words	0.83	0.77	874.5	19.71 (1.42)
HIL + SWL Text	0.79	0.75	409.4	145.0 (74.8)
HIL words + Text	0.82	NA	543.7	79.2 (38.4)
SWL words + Text	NA	0.80	518.4	85.5 (46.0)
HIL Words	.79	0.66	807.0	9.8 (1.0)
HIL Text	.74	0.61	380.1	69.4 (38.4)
SWL Words	.75	0.75	653.0	9.9 (0.73)
SWL Text	.71	0.74	379.4	75.6 (45.9)

All correlations were significant at *p* < 0.001. *N* = 608. BERT large using the second last layer (L23). HIL = Harmony in life; SWL = Satisfaction with life; S = Scale.

Diversity index of Words Input is 2^entropy^, which indicates how many different “types” (i.e. distinct categories) that could theoretically be accounted for by the data.

### Using multiple response formats and questions improves accuracy

Two response formats were significantly more accurate than one response format for the following four relevant combinations of comparisons. The tests compared predictions of the Harmony in life scale based on: Harmony in life word- and text-responses versus only Harmony in life word-responses (*r* = 0.82 versus 0.79, *p* < 0.001) and only Harmony in life text-responses (*r* = 0.82 versus 0.74, *p* < 0.001); as well as predictions of the Satisfaction with life scale scores based on: Satisfaction with life word- and text-responses versus only Satisfaction with life word-responses (*r* = 0.80 versus 0.75, *p* < 0.001) and only Satisfaction with life text-responses (*r* = 0.80 versus 0.74, *p* < 0.001).

Next, we examine how accurately *multiple* word- and/or text-responses from different topics/constructs predict rating scales. Responses from two construct questions produced predictions that were significantly more accurate than predictions from one construct question for all but one comparisons. The significance tests included comparing the Harmony in life scale predictions based on: Harmony in life and Satisfaction with life word-responses versus only Harmony in life word-responses (*r* = 0.83 versus 0.79, *p* < 0.001); and Harmony in life and Satisfaction with life text-responses versus only Harmony in life text-responses (*r* = 0.79 versus 0.74, *p* < 0.001). And comparing the SLWS predictions based on Harmony in life and Satisfaction with life word-responses versus only Satisfaction with life word-responses (*r* = 0.77 versus *r* = 0.75, *p* = 0.039); and Harmony in life and Satisfaction with life text-responses versus Satisfaction with life text-responses (*r* = 0.75 versus 0.74, *p* = 0.111).

## Discussion

### Beyond state-of-the-art and the reliabilities

Language-based assessments analyzed with modern transformer language models that enable contextualized word embeddings yield unprecedented high predictive accuracy of rating scales. Combining responses from both word- and text-responses about Harmony in life and Satisfaction with life yields the highest accuracy, which is significantly higher than previous methods. The predictive accuracy for the Harmony in life scale is even higher than the rating scales’ reliability as it is typically measured and seen as the theoretically highest limit.

These results demonstrate that word- and text-responses contain valuable information in relation to previously validated rating scales, which further emphasizes the significance of evidence supporting language-based assessments. This includes evidence showing that they exhibit higher, or competitive, degrees of validity and reliability when compared with rating scales^10^. This has, for example, been shown when comparing language-based assessments’ and rating scales’ ability to accurately categorize *external stimuli* of pictures depicting facial expressions including sad, happy and contemptuous. Another study revealed a significant positive correlation between theoretically relevant cooperative *behavior* and the language-based assessments of harmony in life (Pearson’s *r* = 0.18; and *r* = 0.35 in participants categorized as prosocials), but not the corresponding rating scale^7^.

### Ability to distinguish well-being dimensions

The high significant predictive accuracies support the validity of both the rating scales and the language-based assessments. Since the word- and text-responses were presented *before* the rating scales (see the Method section), the items composing the rating scale did not influence respondents’ view of the targeted psychological construct. Interestingly, the language response for a specific construct tended to predict its corresponding rating scale the best. The Harmony in life responses predict the Harmony in life scale better than the Satisfaction with life scale; however, this was not true for the Satisfaction with life responses and the Satisfaction with life scale. Further, whereas the Satisfaction with life scale is better predicted by Satisfaction with life responses than Harmony in life responses; the Harmony in life scale is only better predicted by the HIL text-responses rather than the Satisfaction with life text-responses.

Lastly, despite the very strong correlation between the rating scales, it is possible to create a discriminant model that significantly predicts their difference scores. These findings demonstrate that respondents perceive the constructs differently, and are able to describe this with both language and through rating scales. The AI methods demonstrate that individuals show surprising concordance between self-reported rating scales and open-ended questions.

### Contextualized word embeddings

Contextualized, as compared to decontextualized, word embeddings particularly increase the predictive accuracy from text-responses. They substantially increase the predictability of text-responses, which almost reaches the same accuracy as descriptive word-responses (only word-responses for Harmony in life are significantly more predictive than the text-responses, and also with a small effect size). This is an important finding because it opens up the opportunity to make better use of the text-response format.

### Complementary response formats

Comparing the strongest correlation when only using one response (*r* = 0.79, Table 4) versus using all responses (*r* = 0.85, Table 3), demonstrates that adding responses increases the predictive accuracy. The different response formats complement each other in both predictive ability and practical advantages. Descriptive words are less demanding to write (i.e., fewer words to write), text is more natural (i.e., less constrained). Further, changing response formats may promote thinking about the same question in different ways, from different perspectives.

### Comparisons with predictions of subjective states from social media text

Language-based assessments based on direct prompts/questions produce considerably stronger correlations than those derived from individuals’ social media profiles. Research shows that it is also possible to assess individuals’ subjective states of mind by analyzing their social media text (e.g., from Facebook and Twitter). These analyses have been demonstrated to rigorously predict psychological and health related outcomes such as satisfaction with life (*r* = 0.57)^25^, and personality (*r* = 0.31-0.42)^2^. But, language-based assessments based on prompts are different from social media text analyses, as they resemble rating scales in directly asking individuals to communicate their state of mind rather than using naturally occurring data, which potentially explains the current unprecedented high predictive accuracy.

### Limitations, Conclusions and Future Research

This study focuses on examining the relationships between language-based assessments and rating scales; but, it does not compare which of the two are the most valid or reliable. Even though the rating scales have been validated, and used in a wide range of research settings^14,26^, self-reported measures are not objective truth, and future research should compare rating scale and language-based assessments in predicting theoretically relevant behaviors, biological markers etc. Notably, language-based assessments can also be constructed to predict these outcomes directly, and thus assess mental states independent of rating scales. Lastly, the sample included respondents online from the USA only, generalization beyond this should be done with caution.

We show that open-ended, text-responses predict rating scales with unprecedented accuracy. The accuracy is not only significantly higher than previous methods, but higher than or rivaling the typical ways that scales’ reliability are measured–which is normally conceived as the upper theoretical limit^23^. We also show that using only 10 descriptive words can reveal a lot of psychological information, that contemporary language models produce very accurate predictions from analyzing text-responses, and that combining responses increases the predictive accuracy as compared with only using one response. These results provide promising evidence that language, the most natural way of conveying complex psychological traits and states of mind, can be quantified to improve and modernize current research methods and improve clinical practices. We envision that these methods are applicable for widespread use in scientific research, including fields such as psychology, neuroscience, or medicine. Hence, these findings suggest an avenue for modernizing self-report human assessment and ultimately opening doors to a greater understanding of the human condition.

## Methods

### Participants

The data used here is by convenience pooled from three previously published studies that received ethical approval from the Regional Ethics Board in Lund, and adhered to Swedish laws (Study 3: *N* = 92; Study 4: *N* = 303; Study 5: *N* = 296)^10^. Participants were recruited using Mechanical Turk (www.mturk.com). Fifty-six participants were excluded for not answering the control items correctly, which were for Study 3: N = 13, Study 4, N = 24, and Study 5, N = 19. Out of the remaining 691 participants, an additional 79 were excluded for not reporting USA as nationality; and 4 participants were excluded for not answering all four open-ended questions. The final sample comprised 608 participants from the USA (359 females, 249 males, 0 others), with a mean age of 35.0 (*SD* = 12.88, range = 18–74) years. Participants’ perceived household economic situation ranging from “1 = Our income does not cover our needs, there are great difficulties” to “7 = Our income covers our needs, we can save” had a reported mean of 4.44 (SD = 1.96).

### Instruments

*Open-ended questions for harmony in life (*i.e., “Overall in your life, are you in harmony or not?”) *and satisfaction with life (*i.e., “Overall in your life, are you satisfied or not?”), were coupled with instructions to either answer with 10 descriptive words or a text-response.

*The Harmony in Life Scale*^17^ measures Harmony in life with five items (e.g., “My lifestyle allows me to be in harmony”), coupled with closed-ended rating scales ranging from “1 = Strongly Disagree” to “7 = Strongly Agree”.

*The Satisfaction with Life Scale*^16^ measures Satisfaction with life with five items (e.g., “I am satisfied with my life”) coupled with the same rating scales alternatives as the Harmony in life scale.

For more details of measures see SM.

### Procedure

The studies followed Swedish law and received ethical approval from the Regional Ethical Committee in Lund, Sweden (2014/396). Participants were first informed about the general purpose of the studies and their right to withdraw at any time, that their responses were confidential and that they did not involve collecting personal identifiable information – informed consent was then obtained from all participating participants. First, participants were asked to answer the open-ended questions, which were presented in random order. Subsequently, the rating scales were presented in random order. The demographic questions were presented last, followed by debriefing. The mean time to complete the studies were 14.58 (SD = 9.02), 16.44 (SD = 19.48) and 20.02 (SD = 10.31) minutes for study 3, 4 and 5, respectively^10^.

### Analytic method

The text analyses were carried out in *Text*^27^ (version 0.9.11), which is an R-package^28^ specialized in enabling social scientists to use state-of-the-art natural language processing and machine learning. In short, the current analyses involve applying word embeddings from pre-trained language models to quantify the word- and text-responses; and then training the word embeddings to predict the rating scales using regression.

#### Pre-trained word embeddings

To train high-quality word embeddings requires large amounts of text data; and when that is not available it is possible to use a general-purpose language representation model built on other text data. This is known as pre-training. We will compare bidirectional contextual and context-free pre-trained word embeddings. Bidirectional contextual word embeddings are influenced by other words in a text. In “She looked at the bank account”, the embedding of “bank” is *also* influenced by the previous *and* following context (i.e., “She looked at the … account”). The primary bidirectional pre-trained model used here comes from Google’s open-source model called BERT (“bert-large-uncased”; henceforth BERT; Devlin et al., 2019). BERT represents tokens (c.f. words) with 24 layers comprising 1024 dimensions each. Only the second to last layer was used based on research demonstrating that this layer yields reliable performances for document- and human-level predictions^12,29^. The contextualized embeddings will be compared with context-free embeddings by letting the BERT model only see one word at a time (1 word/document), and using the context-free Latent Semantic Analysis based model from Kjell et al. (2019).

The BERT-large (“bert-large-uncased”) and Latent Semantic Analysis models are also compared with BERT-base^7^ (“bert-base-uncased”) and DistilBert^23^ (“distilbert-base-uncased”). These models are based on text retrieved from Wikipedia and a Book corpus. BERT-large comprises 24 layers, where we used layer 23 for the analyses; the BERT-base model comprises 12 layers, where we used layer 11 for the analyses; and DistillBERT has 6 layers, where we used layer 5. For more details about the creation of the BERT model see^7^.

#### Training word embeddings to rating scales

To examine the relationship between word- and text-responses with rating scales, the word embeddings dimensions of the responses are used as predictors in ridge multiple regression^30^ to predict the rating scale scores. Training is employed using tenfold cross-validation, where the training set is further split for analysis (75% of the training data is used to create models with different penalties) and assessment (25% used to evaluate the different models). The prediction accuracies are evaluated with Pearson correlation between observed and predicted scores.

The training sets were stratified according to the outcome (y), using 4 bins to stratify over. Further, the search grid for the penalty in ridge regression ranged from 10^−16^–10^16^, with increases of times 10. For more details see^16^. These ranges were based on a wide range of empirical evidence from applications of the DLATK package^31^ which has applied the same models for Python rather than R.

Our models use a convex optimization on top of pre-trained transformers (shown to be ideal for person-level assessments when having N people < 1000^32^). Therefore, optimization epochs are run until convergence, which is guaranteed. Advanced users may wish to leverage fine-tuning aspects in which maximum epochs and early stopping criteria which are exposed through the libraries bindings with the HuggingFace transformers package^33^. Future work will provide a guide for such an approach.

#### Significance testing the prediction accuracy between models

To test the difference between two prediction models of the same outcome, we first compute the error for each prediction (i.e., y-$\widehat{y}$), and then use a paired sample t-test to compare whether the errors differ between the two models. To test two prediction models of different outcomes (e.g., comparing a model that predicts the Harmony in life scale with one predicting the Satisfaction with life scale) a bootstrapped procedure was used. We used a monte-carlo simulation^34,35^ whereby bootstrapped resampling was used to create a distribution of accuracies for each model. Subsequently, the overlap of the two bootstrapped distributions were compared.

#### Reliability of the to-be-predicted measure as the upper limits of prediction accuracies

It is important to consider the upper limits of how accurate rating scales can be predicted. Statistically, an observed correlation between two measures is *not only* influenced by the relation between the latent traits, but also the reliability of the measures (see the Attenuation Correlation Coefficient^23,36^); where the reliabilities of the measures limit the upper bound of the correlational strength that may be found. For example, the measurement of weight and height can be measured with a reliability of near 1.0, which means that a correlation of *r* = 0.4, represents 0.4 out of an upper limit of *nearly* 1.0. In contrast, self-report measures of psychological constructs tend to exhibit considerably lower reliabilities, which thus lowers the possible upper limit. Measures of psychological constructs that are considered well-constructed often display reliabilities around *r* = 0.8. Hence, an *r* = 0.7 between actual and predicted scores of such measures can be seen as 0.7 out of the upper limit of 0.8. Therefore, we compare correlations between predicted and observed scores with the rating scales’ reliability as measured by their test–retest reliabilities (i.e., across time as retrieved from previous research), the rating scales’ corrected item-total correlations (i.e., the mean of the Pearson correlation between each item and the total score of all other items in the scale) and inter-item correlation average (i.e., the mean of the Pearson correlations among all individual items). We used reliability measures based on Pearson product-moment correlation instead of other reliability metrics, such as Cronbach's alpha and McDonald’s omega (which are reported in the supplement material), so that it is directly comparable to the Pearson-correlation between our predicted scores and the observed scores.

#### Information theory

The Diversity Index based on Shannon Entropy^37^ (i.e., 2^entropy^) is used to measure how much information a response format comprises. This is a key measure in machine learning as it indicates how much information the algorithms have at their disposal to learn.

#### Cutoffs

Alpha was set to 0.05. All correlations were computed as Pearson product-moment correlation coefficients (r). Correlations of 0.2-0.39 are interpreted as weak, 0.40-0.59 as moderate, 0.6-0.79 as strong and 0.8–1.0 as very strong.

### R-References

Analyses were carried out in RStudio^38^, and included using the following packages: text^27^, tidyverse^39^, entropy^40^, stringr^41^, tidyr^42^, Hmisc^43^, data.table^44^, car^45^, rsample^46^, and psych^47^.

## Supplementary Information

Supplementary Information.
