# Supplementary material for: Natural language analyzed with AI-based transformers predict traditional subjective well-being measures approaching the theoretical upper limits in accuracy
Source: Sci Rep. 2022 Mar 10;12:3918. doi: 10.1038/s41598-022-07520-w (PMC8913644; doi:10.1038/s41598-022-07520-w)
Supplement: Supplementary file 1 — Supplementary Information. [file 41598_2022_7520_MOESM1_ESM.docx]

Supplemental Online Materials

Response Formats and Algorithms

Answering different response formats or multiple but related construct questions are hypothesized to increase predictive validity compared to answering a single response format or construct question. This hypothesis is not only based on the fact that more information is available, but also on the assumption that each question enables respondents to change perspectives, providing greater opportunities to elaborate on the to-be-measured construct. From a statistical point of view, multiple inputs may also allow the model to conduct paired comparisons of the data, allowing control for some idiosyncratic response patterns of the assessed individual.

Method

*Material*

Below are two complete examples of the instructions for the word- and text-response questions for the harmony in life question^1^.

“Please answer the question by writing 10 descriptive words below that indicate whether you are in harmony or not. Try to weigh the strength and the number of words that describe if you are in harmony or not so that they reflect your overall personal state of harmony. For example, if you are in harmony then write more and stronger words describing this, and if you are not in harmony then write more and stronger words describing that.
Write descriptive words relating to those aspects that are most important and meaningful to you. Write only one descriptive word in each box.”

“Please answer the question by writing at least a paragraph below that indicates whether you are in harmony or not. Try to weigh the strength and the number of aspects that describe if you are in harmony or not so that they reflect your overall personal state of harmony. For example, if you are in harmony then write more about aspects describing this, and if you are not in harmony then write more about aspects describing that.
Write about those aspects that are most important and meaningful to you.”

For the questions allowing text-responses, there was a minimum response limit of 100 characters. If a respondent wrote fewer characters, the following message was presented: “Please write a little bit more. Please enter at least 100 characters, which is approximately 20 words.”

The Harmony in Life Scale^2^ yielded a Cronbach’s alpha of .94, and a McDonald’s omega hierarchical of .91 in the current dataset.

The Satisfaction with Life Scale^3^ produced a Cronbach’s alpha of .93 and a McDonald’s omega hierarchical of .89 in the current dataset.

*Control items* such as “Answer ‘disagree’ on this question”, where interspersed among the rating scales items; one control item per rating scale. Participants who did not answer all correctly were excluded from the analyses, as this type of attention checks have been demonstrated to increase the quality of datasets ^4^.

*Demographics* included questions concerning age, gender and perceived economic situations. The perceived household economic situations item asked: “Does the total income of your household allow you to cover your needs?”, which were coupled with 7 closed-ended alternatives ranging from “1 = Our income does not cover our needs, there are great difficulties” to “7 = Our income covers our needs, we can save”.

Analytic Method

The context-free model provides one word embedding for every word in the pre-trained model. That is, for context-free representations, the word “bank” has the same representation in “bank account” and “river bank”. The context-free pre-trained model in this study comes from Semantic Excel (English Space 1)^7,8^; the model has been produced using Latent Semantic Analysis^9^ applied to text from the Google N-gram database (<https://books.google.com/ngrams>).

To create a word embedding that represents all words in a word-response or all text in a text-response, the word embeddings of each word are aggregated using the mean of each dimension of the word embedding.

Note that since the advent of transformers and BERT there have been many more models developed such as RoBERTA^10^, ALBERT^11^ as well as multilingual models such as mBERT, which can represent more than 100 languages. All these models and more are available to use in *Text*^12^.

*Statistical Cutoffs.* Cronbach’s alpha and omega were considered good at .70 or above.

Results

Descriptions of the numeric variables

Table SM1 shows descriptive statistics of the rating scale variables; which are normally distributed, where all have a skew and kurtosis within ±2. Table SM2 presents the correlations among rating scales, where the Harmony in life scale and the Satisfaction with life scale correlate very strongly (Pearson *r*=.85. *p*<.001; see Table SM2)

| Table SM1.  Descriptive statistics of numeric variables | | | | | |
| --- | --- | --- | --- | --- | --- |
| **Variable** | **Mean** | **SD** | **Min-Max** | **Skew** | **Kurtosis** |
| **HILS** | 25.18 | 8.03 | 5 - 35 | -0.83 | -0.37 |
| **SWLS** | 22.85 | 8.44 | 5 - 35 | -0.58 | -0.72 |
| **Age** | 35.61 | 12.88 | 18 - 74 | 0.81 | -0.40 |
| **Economy** | 4.44 | 1.96 | 1 - 7 | -0.33 | -1.08 |
| Notes. HILS = Harmony in life scale; SWLS = Satisfaction with life scale; Economy = Perceived economy. N=608 | | | | | |

| Table SM2.  Pearson correlations among numeric variables | | | |
| --- | --- | --- | --- |
|  | **1.** | **2.** | **3.** |
| **1. HILS** |  |  |  |
| **2. SWLS** | .85*** |  |  |
| **3. Age** | .06 | .01 |  |
| **4. Economy** | .47*** | .47*** | -.03 |
| Note. HILS = Harmony in life scale; SWLS = Satisfaction with life scale; Economy = Perceived economy; *** = p < .001.N = 608 | | | |

Language-based Assessments as Accurate as Rating Scale Reliability

Table SM3 shows predictive performance of a variation of language models including Bert base and DistillBERT.

| *Table SM3. Extended version of Table 2 Including More Types of Word Embeddings.*  *Comparison of Using Contextualized versus Decontextualized Word Embeddings for Individual Word- and Text-Responses* | | | | | |
| --- | --- | --- | --- | --- | --- |
|  | **Word Embeddings** | **Text** | | **Words** | |
|  |  | **HIL** | **SWL** | **HIL** | **SWL** |
|  |  | **HILS** | **SWLS** | **HILS** | **SWLS** |
| **Context** | **BERT** | .74 | .74 | .79 | .75 |
|  | **BERT base** | .73 | .71 | 78 | .75 |
|  | **Distil Bert** | .69 | .69 | .78 | .75 |
| **No context** | **BERT 1 word docs** | .54 | .59 | .78 | .75 |
|  | **BERT layer 0** | .58 | .59 | .79 | .74 |
|  | **Latent Semantic Analysis^1^** | .47 | .46 | .75 | .72 |
|  | **Latent Semantic Analysis** | .42 | .36 | .68 | .54 |
| Note. All correlations were significant at *p* < .001. *N* = 608. BERT using the second last layer (L23).  HIL = Harmony in life; SWL = Satisfaction with life; S = Scale.  Latent Semantic Analysis based on Google 5-gram, 512 dimensions.  ^1^ = number of dimensions were optimized as described in^1^ (i.e., based on previous state-of-the-art). | | | | | |

Discussion

Limitations of Reliabilities

The reliability of a scale provides limits on how well they can be predicted. That is, the possible upper limit of the prediction accuracy – how well the assessment captures the intended outcome, is theoretically bounded due to imperfect reliability of the existing to-be-predicted measure. Reliability measures typically used for psychometrics are, however, not always perfect. For example, considering that subjective well-being measures possible changes over time, the test-retest reliability is also less than perfect (1.0) due to its valid change over time. Importantly though, the Satisfaction with life scale and the Harmony in life scale measure the individuals’ *overall/global* assessment of their lives, which is known to be stable over time^13^, only changing slowly or due to rare larger events that are less likely to occur over shorter periods of, e.g., around 1-2 months.

Similarly, in the construction of measures for psychological constructs, the aim is often to capture the richness of more complex constructs and to include several items with different wordings to be able to create a total score that can balance out measurement errors. As a result, inter-item and item-total correlation averages should typically not yield a correlation of 1.0. For the purposes of this study, both inter-item and item-total correlation averages can be seen to be especially relevant though. This is because both the Harmony in life scale and the Satisfaction with life scale are relatively short scales, comprising only five items each that are all aimed to capture the most central aspects of the constructs. As a result, this is thus creating relatively high inter-item and item-total correlation averages, and thus provides a considerably high reliability measure.

In the absence of one perfect measure, it is in psychometrics instead common to use several different measures. This is achieved in this study by using the three discussed reliability measures, which may be seen as proxies for the true reliability of the scales.

Further, the current study does not examine the importance for predictability in term of larger amount of responses; although research show that the number of descriptive words does not appear to increase the predictability when reaching 9-10 words^1^; this has not been examined for text-responses (and this particular data set is not appropriate to test this because participants have not written sufficient amounts of text).

R-references

Analyses were carried out in RStudio ^14^, and included using the following packages: tidyverse ^15^, entropy ^16^, stringr ^17^, tidyr ^18^, Hmisc ^19^, data.table ^20^, car ^21^, rsample ^22^, and psych ^23^.

References

1. Kjell, O. N., Kjell, K., Garcia, D. & Sikström, S. Semantic measures: Using natural language processing to measure, differentiate, and describe psychological constructs. *Psychol. Methods* **24**, 92 (2019).

2. Kjell, O. N. E., Daukantaitė, D., Hefferon, K. & Sikström, S. The Harmony in Life Scale Complements the Satisfaction with Life Scale: Expanding the Conceptualization of the Cognitive Component of Subjective Well-Being. *Soc. Indic. Res.* **126**, 893–919 (2016).

3. Diener, E., Emmons, R. A., Larsen, R. J. & Griffin, S. The satisfaction with life scale. *J. Pers. Assess.* **49**, 71–75 (1985).

4. Oppenheimer, D. M., Meyvis, T. & Davidenko, N. Instructional manipulation checks: Detecting satisficing to increase statistical power. *J. Exp. Soc. Psychol.* **45**, 867–872 (2009).

5. Devlin, J., Chang, M.-W., Lee, K. & Toutanova, K. BERT: Pre-training of Deep Bidirectional Transformers for Language Understanding. in *Proceedings of the 2019 Conference of the North American Chapter of the Association for Computational Linguistics: Human Language Technologies, Volume 1 (Long and Short Papers)* 4171–4186 (Association for Computational Linguistics, 2019). doi:10.18653/v1/N19-1423.

6. Sanh, V., Debut, L., Chaumond, J. & Wolf, T. DistilBERT, a distilled version of BERT: smaller, faster, cheaper and lighter. *ArXiv Prepr. ArXiv191001108* (2019).

7. Sikström, S., Kjell, O. N. E. & Kjell, K. *Semantic Excel: An Introduction to a User-Friendly Online Software Application for Statistical Analyses of Text Data*. https://osf.io/z9chp (2018) doi:10.31234/osf.io/z9chp.

8. Sikström, S., Kjell, O. N. E. & Kjell, K. SemanticExcel.com: An Online Software for Statistical Analyses of Text Data Based on Natural Language Processing. in *Statistical Semantics: Methods and Applications* (eds. Sikström, S. & Garcia, D.) 87–103 (Springer International Publishing, 2020). doi:10.1007/978-3-030-37250-7_6.

9. Landauer, T. K. & Dumais, S. T. A solution to Plato’s problem: The latent semantic analysis theory of acquisition, induction, and representation of knowledge. *Psychol. Rev.* **104**, 211–240 (1997).

10. Liu, Y. *et al.* Roberta: A robustly optimized bert pretraining approach. *ArXiv Prepr. ArXiv190711692* (2019).

11. Lan, Z. *et al.* Albert: A lite bert for self-supervised learning of language representations. *ArXiv Prepr. ArXiv190911942* (2019).

12. Kjell, O., Schwartz, H. A. & Giorgi, S. Text: an R-package for analyzing and visualizing human language using natural language processing and deep learning. (2021).

13. Lucas, R. E., Freedman, V. A. & Cornman, J. C. The short-term stability of life satisfaction judgments. *Emot. Wash. DC* **18**, 1024–1031 (2018).

14. RStudio Team. *RStudio: Integrated Development Environment for R*. (RStudio, PBC., 2020).

15. Wickham, H. *et al.* Welcome to the Tidyverse. *J. Open Source Softw.* **4**, 1686 (2019).

16. Hausser, J. & Strimmer, K. Entropy: estimation of entropy, mutual information and related quantities. R package version 1.2. 1. *See Httpstrimmerlab Org* (2014).

17. Wickham, H. *stringr: Simple, Consistent Wrappers for Common String Operations*. (2019).

18. Wickham, H. & Henry, L. *tidyr: Tidy Messy Data*. (2020).

19. Jr, F. E. H., Dupont, with contributions from C. & others, many. *Hmisc: Harrell Miscellaneous*. (2020).

20. Dowle, M. & Srinivasan, A. *data.table: Extension of `data.frame`*. (2019).

21. Fox, J. & Weisberg, S. *An R companion to applied regression*. (Sage publications, 2018).

22. Kuhn, M., Chow, F. & Wickham, H. *rsample: General Resampling Infrastructure*. (2020).

23. Revelle, W. *psych: Procedures for Psychological, Psychometric, and Personality Research*. (Northwestern University, 2019).
